# Enhancing Veteran Community Reintegration Research (ENCORE): Protocol for a Mixed Methods and Stakeholder Engagement Project

**DOI:** 10.2196/42029

**Published:** 2023-03-14

**Authors:** Karen Besterman-Dahan, Bridget Hahm, Margeaux Chavez, Jacquelyn Heuer, Christine Melillo, Jason Lind, Christina Dillahunt-Aspillaga, Lisa Ottomanelli

**Affiliations:** 1 Research and Development Service James A Haley Veterans Hospital and Clinics Tampa, FL United States; 2 Department of Anthropology College of Arts and Sciences University of South Florida Tampa, FL United States; 3 Department of Rehabilitation & Mental Health Counseling University of South Florida Tampa, FL United States

**Keywords:** veteran, community, community reintegration, stakeholder engagement, veterans community integration, veteran health administration, knowledge translation, research promotion, veterans association policy, policy

## Abstract

**Background:**

Veteran community reintegration (CR) has been defined as participation in community life, including employment or other productive activities, independent living, and social relationships. Veteran CR is a Veterans Health Administration priority, as a substantial proportion of veterans report difficulties with veteran CR following discharge from military service.

**Objective:**

Enhancing Veteran Community Reintegration Research (ENCORE) is a project funded by Veterans Health Administration’s Health Service Research and Development Service. The goal of ENCORE is to maximize veteran and family reintegration by promoting innovative research and knowledge translation (KT) that informs and improves equitable Department of Veterans Affairs (VA) policies, programs, and services. Overall, 2 strategic objectives guide ENCORE activities: mobilize veteran CR research and promote innovation, relevance, and acceleration of veteran CR research and KT.

**Methods:**

ENCORE uses a mixed methods and stakeholder-engaged approach to achieve objectives and to ensure that the KT products generated are inclusive, innovative, and meaningful to stakeholders. Project activities will occur over 5 years (2019-2024) in 5 phases: plan, engage, mobilize, promote, and evaluate. All activities will be conducted remotely owing to the ongoing COVID-19 pandemic. Methods used will include reviewing research funding and literature examining the gaps in veteran CR research, conducting expert informant interviews with VA program office representatives, and assembling and working with a Multistakeholder Partnership (MSP). MSP meetings will use external facilitation services, group facilitation techniques adapted for virtual settings, and a 6-step group facilitation process to ensure successful execution of meetings and accomplishment of goals.

**Results:**

As of December 2022, data collection for ENCORE is ongoing, with the team completing interviews with 20 stakeholders from 16 VA program offices providing veteran CR–related services. ENCORE developed and assembled the MSP, reviewed the VA funding portfolio and veteran CR research literature, and conducted a scientific gap analysis. The MSP developed a veteran CR research agenda in 2021 and continues to work with the ENCORE team to prepare materials for dissemination.

**Conclusions:**

The goal of this program is to improve the impact of veteran CR research on policies and programs. Using a stakeholder-engaged process, insights from key stakeholder groups are being incorporated to set a research agenda that is more likely to result in a relevant and responsive veteran CR research program. Future products will include the development of an effective and relevant dissemination plan and the generation of innovative and relevant dissemination products designed for rapid KT.

**International Registered Report Identifier (IRRID):**

DERR1-10.2196/42029

## Introduction

### Overview

Enhancing Veteran Community Reintegration Research (ENCORE) is a 5-year project funded by Veterans Health Administration (VHA) in July 2019. The goal of ENCORE is to improve the Department of Veterans Affairs (VA) policies, programs, and services related to veteran community reintegration (CR), which is a VHA priority [1]. Veteran CR has often been defined as participation in community life, including employment or other productive activities, independent living, and social relationships [2]. ENCORE will achieve its goal by (1) mobilizing veteran CR research and (2) promoting innovation, relevance, and acceleration of veteran CR research and knowledge translation (KT). The ENCORE team developed a road map establishing how these 2 strategic objectives will guide activities to meet the project’s impact goal. This protocol paper outlines this road map, including the methods that will be used to determine the current state of veteran CR research, engage stakeholders in shared decision-making using a Multistakeholder Partnership (MSP), and communicate with diverse audiences about veteran CR research needs and priorities. This protocol paper is intended to inform researchers and other professionals on underused methods to engage multiple stakeholders while prioritizing research, program, and policy needs of specific populations.

Veteran CR is a key determinant of veteran health, functioning, and quality of life [3]. A substantial proportion of veterans report difficulties with veteran CR following discharge from military service including poor social and family relationships, unemployment, financial strain, homelessness, and decreased physical and mental health [4,5]. These difficulties are further compounded for veterans with disabilities including the approximately 4.9 million veterans having a service-connected disability [6,7]. Successful veteran CR for all populations emphasizes engagement with community, peers, and family. VHA strategic initiatives outline the importance of establishing and enhancing community relationships that affect and promote veteran CR health [4,5].

ENCORE focuses on two critical domains of veteran CR: (1) work and other productive activities and (2) social relationships and activities (eg, interaction with family members and friends, parental or marital relationships, and marital issues). Employment or other meaningful and productive activity is directly related to income stability, independence, health, and a sense of meaning and purpose in life [8-10]. Evidence demonstrates that the strength and quality of one’s social relationships and perceived social support also play key roles in health and well-being [8,11].

### Background

#### KT Strategies

KT, defined “as a dynamic and iterative process that includes the synthesis, dissemination, exchange and ethically sound application of knowledge to improve health, provide more effective health services and products, and strengthen the health care system” [12], is a key component that underpins ENCORE’s strategic objectives. Knowledge creation, distillation, and dissemination alone are not sufficient to ensure evidence-informed decision-making [12]. KT is widely used to ensure that decision makers at all levels of the health system (eg, consumers, patients, practitioners, managers, and policy makers) are aware of and can access and use research evidence to inform health-related decision-making [13-16]. Critical considerations suggest that KT should include all stakeholder groups (eg, patients, health care providers, and policy makers) in the actual process of using knowledge to facilitate decision-making [17,18]. Strategies for KT must vary according to the target audience (eg, researchers, clinicians, policy makers, and public) and the type of knowledge being translated (ie, clinical, biomedical, and policy-related) [18-20].

#### Stakeholder Engagement

Stakeholder engagement in KT is increasingly recognized as important [21,22]. Stakeholders are defined as “individuals, organizations or communities that have a direct interest in the process and outcomes of a project, research or policy endeavor” [23]. The Institute of Medicine, Agency for Healthcare Research and Quality, James Lind Alliance, and Patient Centered Outcomes Research Institute have all developed frameworks for systematically including patients in research, from topic generation to dissemination of results [24-28]. The involvement of relevant stakeholders in all phases of research has been called authentic stakeholder engagement [29].

Authentic engagement includes stakeholders as full partners in setting research priorities; forming research questions; and shaping the design, funding, conduct, and dissemination of studies. Authentic stakeholder engagement must be built on a foundation of trust between researchers and stakeholders [29,30]. In their review of the literature in 2018, Boaz et al [21] described 3 broad categories of important requirements for building this trust and authentically engaging stakeholders: organizational, value, and practice requirements. At the organizational level, researchers should begin by embedding stakeholder engagement into the frameworks of their projects and then allocating resources to support not only the engagement activities but also the evaluation of engagement and rewarding successes [21]. Next, researchers should foster shared values among researchers and stakeholders, including understanding roles, expectations, and activities and encouraging commitment to sustained and continuous engagement [21]. Finally, Boaz et al [21] identified 5 practices focused on ensuring that stakeholder engagement activities are flexible and systematically enacted and that stakeholder feedback is collected and applied to improvement efforts.

Within VHA, the use of stakeholder engagement methods has steadily increased in the past 2 decades [22]. Methods including the use of advisory councils, veteran engagement groups (VEGs), community-based participatory research, action research, patient-centered research, and human-centered design have become popular within VHA [22]. There is still a need to identify ways to measure and evaluate the quality of stakeholder engagement and its outcomes [31-33]. However, the impacts of stakeholder engagement on research have been documented in the literature both within the VHA [22] and within the broader scientific community [21]. Partnerships among those who produce research and those who use it are likely to enhance the relevance of research and facilitate its use [30]. An engaged stakeholder-driven process to generate and prioritize a research agenda increases the relevance and utility of sustainable interventions and services [34].

#### The Need to Assess Gaps in Current CR Research

In 2010, a Rehabilitation Research and Development (RR&D) state-of-the-art (SOTA) conference was convened on *issues in defining and measuring veteran CR* [9]. At the SOTA conference, researchers discussed ways to improve the measurement of outcomes for veteran CR, including outlining goals to accomplish this, based on current gaps in the literature.

Since the SOTA conference, the bulk of veteran CR research has focused on assessing CR among individuals with mental health conditions, including projects focusing specifically on posttraumatic stress disorder (PTSD), traumatic brain injury (TBI), and polytrauma [11,35-51]. Some of the SOTA goals have received limited attention, such as the need for more comprehensive measurement approaches for veteran CR. Although new measurements were in development at the time of the SOTA conference (eg, Military to Civilian Questionnaire [M2CQ], [52]; Community Reintegration of Injured Service Members [CRIS], [9]; and Community Reintegration of Injured Service Members Computer Adaptive Test [CRIS-CAT], [9]), other measures are yet to be developed and validated within the VA [52-55]. Moreover, little research has been conducted to complete head-to-head comparisons of CR measurements and instruments [55,56].

Another goal that has received limited attention is the need to measure veteran CR across the life span. Given that veteran CR is often a cyclical process that occurs throughout the lifetime of a veteran, this is an aspect of veteran CR research that requires more attention in coming years [51,57]. Similarly, the effect of veteran CR on society also needs to be studied more, as the CR struggles of veterans may have ripple effects within their communities [58-60].

Since Resnik et al [9] published the SOTA proceedings in 2012, it has become a seminal reference in veteran CR research, particularly for VA-funded research. In support of the goal of this project to enhance the impact of VA research for improving veteran CR, the SOTA conference became a natural point of reference. It became a starting point for the need to assess where the current gaps in the science of veteran CR research remain.

## Methods

### Design and Overview

**Figure 1:**
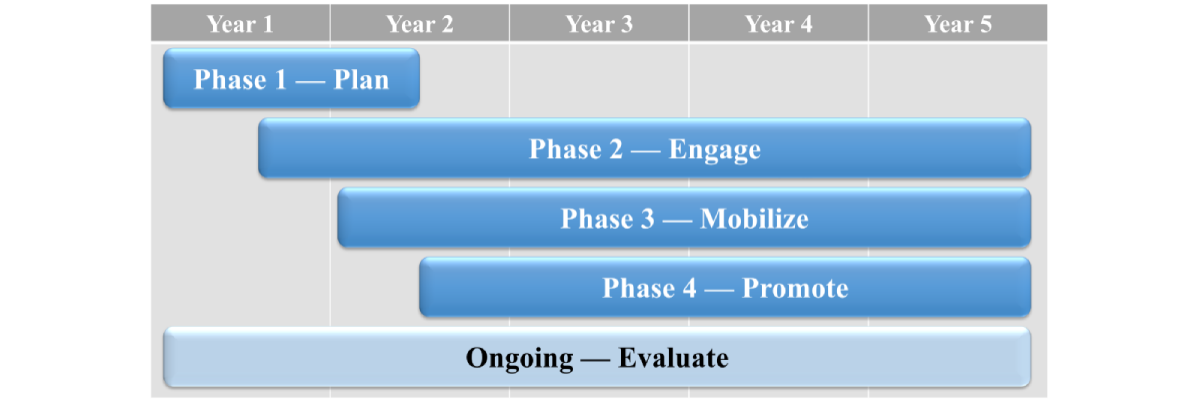
Timeline for the Enhancing Veteran Community Reintegration Research project’s 5-year road map.

The ENCORE project’s 5-year road map contains five phases of activities designed to accomplish project objectives (Figure 1): (1) plan, (2) engage, (3) mobilize, (4) promote, and (5) evaluate. This project uses primary and secondary data and stakeholder engagement activities. The methods used during these phases will be described in detail in the following sections.

### Phase 1 (Plan): Review of Research and Assemble the MSP

#### Review of Research

The findings of the 2010 RR&D SOTA conference on *issues in defining and measuring veteran CR* will be used as a starting point for the review of veteran CR research [9]. At the SOTA conference, researchers discussed ways to improve the measurement of outcomes for veteran CR, including discussions of how veteran CR should be measured, challenges that arise when measuring veteran CR, and recommendations for improving veteran CR measurement. In addition, recommendations were made for future research and policies on veteran CR.

For VA-funded veteran CR grants, the review will focus on grants funded by the VA’s Office of Research and Development and specifically through the following service lines: Health Services Research and Development, RR&D, and Clinical Science Research and Development. For non–VA-funded veteran CR grants, the review will identify grants sponsored by the National Institutes of Health, Department of Defense, and private foundations (eg, Robert Wood Johnson Foundation). Searches will be conducted within VA research databases (including Office of Research and Development) and the National Institutes of Health Reporter and PubMed databases. Research that examined veteran CR issues will be inputted into a Microsoft Excel (Microsoft Corp) tracking sheet. Additional searches for veteran CR research projects will be conducted within Google Scholar for investigators who were known to be linked to VA veteran CR research (VA principal investigators) to identify any missing studies. As the identified literature is reviewed, the reference lists will also be reviewed for relevant publications that did not appear in the literature searches.

The VA Evidence Synthesis Program (ESP) is a resource program that provides timely and accurate syntheses of targeted health care topics of importance to clinicians, managers, and policy makers, as they work to improve the health and health care of veterans [61]. The project team will collaborate with the VA ESP to conduct a search for published literature on veteran CR research focusing on the following question: What studies have examined interventions for promoting veteran CR (eg, reintegration into work, school, or other productive activities or reintegration into social relationships) among Operation Enduring Freedom, Operation Iraqi Freedom, and Operation New Dawn veterans with PTSD, TBI, spinal cord injury (SCI), or polytrauma? This ESP review focused on this selected group of veterans for both pragmatic and scientific reasons. Most of the studies reviewed for the SOTA conference consisted of studies conducted in rehabilitation populations, which include veterans with diagnoses such as PTSD, TBI, SCI, and polytrauma. The ESP review will enable a targeted review of interventions for these populations that have been published since the SOTA conference was conducted. In addition, focusing the ESP review on these populations will enable the team to conduct a more comprehensive review of the veteran CR literature, which includes work involving the military to civilian transition and other CR-related topics. Finally, the general review of the veteran CR literature will focus on the following questions: (1) What are the gaps in evidence that could be informed by future research? and (2) What are the recommendations for future research? This review will include search results from PubMed, VA research databases, Google, and Google Scholar and will focus on published peer-reviewed journals and the *gray literature* (eg, congressional reports and technical reports).

#### Assemble the MSP

The MSP was strategically assembled to set a veteran CR research agenda for the VA. An MSP is a collaboration across multiple sectors with vested interests in an issue [22,34,62].

##### Sampling

###### Overview

Members of the MSP will have experience in veteran CR and a vested interest in advancing impactful research in this field. The MSP will comprise (1) VA CR program leaders; (2) representatives from community-based veteran service organizations (VSOs); (3) veterans, their families, and caregivers; and (4) researchers with expertise in veteran CR. ENCORE can provide stipends to 16 non-VA employee members of the MSP. Microsoft Excel will be used to build a database of potential MSP members. The database will be organized according to member type. A nonexhaustive list of potential MSP members will be added to the database using a set of inclusion criteria (Textbox 1).

**Inclusion criteria for the Multistakeholder Partnership member groups.**

**Inclusion criteria for Department of Veterans Affairs community reintegration (CR) program leaders**

- Program office goals and services align with at least one of the primary veteran CR domains of focus: (1) work and other productive activities and (2) social relationships and activities
- Program office supports either one of the following:
  Priority populations that are at great risk for veteran CR challenges
  Priority populations that are underrepresented in veteran CR research
  Services that address specific diagnoses that place veterans at great risk for veteran CR challenges
  Nominated by operational partner during expert interview

**Inclusion criteria for community-based veteran service organizations**

- Organization passes Department of Veterans Affairs due diligence criteria
- Organization has specific veteran-focused programming that addresses primary veteran CR domains of focus: (1) work and other productive activities or (2) social relationships and activities
- Organization supports priority populations that are at great risk for veteran CR challenges or those that are underrepresented in veteran CR research
- Organization is not engaged in lobbying, for-profit activities, or sponsoring research
- Organization has transparent financial and affiliate information

**Inclusion criteria for veterans, their families, and caregivers**

- Expressed interest in veteran CR
- Commitment to collaborate with Multistakeholder Partnership by sharing CR experiences and abiding by rules of group engagement
- Willing to participate in the Multistakeholder Partnership for 1-2 years

**Inclusion criteria for CR researchers**

- Demonstrated expertise in veteran CR that resulted in the development of a model, instrument, policy, collaboration, and so on, related to veteran CR
- Demonstrates a broad and historical view of veteran CR
- Work aligns with at least one of the primary veteran CR domains of focus: (1) work and other productive activities and (2) social relationships and activities
- Does not have another role in the Enhancing Veteran Community Reintegration Research project
- Willing to participate for 1-2 years

###### VA CR Program Office Leaders

Expert informant interviews with this group will be used to recruit VA veteran CR program office leaders into the MSP. Interviewees will be asked to participate in the MSP or nominate someone to represent the program office on their behalf.

###### Representatives From Community-Based VSOs

As the scope of ENCORE was on veteran CR research agenda to target future research and increase the impact of existing research, recruitment focused on national organizations and programs that still have critical contact with veterans who are not enrolled in or using VA services. To build the list of potential non-VA community veteran CR program leaders, members of the research team will search for VSOs using the following resources:

1. The national directory of VSOs assembled by the VA
2. The VA’s congressionally chartered National Advisory Committee list of VSOs
3. The VA Office of Community Engagement’s current list of VA-partnered community organizations
4. Internet search engines, using combinations of the following terms: “Veteran,” “community,” “reintegration,” “integration,” “inclusion,” “return,” “nonprofit,” and “service”

In addition, the ENCORE team will ask for VSO nominations from the James A Haley Research Service VEG.

To refine the database, ENCORE will assess the appropriateness of partnering with each organization with the VA’s internal *Non-Profit Organization Due Diligence Worksheet* [63] to ensure that the nonprofit organization serves veterans with transparency and integrity (ie, clear mission, goals, and history; available list of board and staff members; available list of services, products, and clientele; verified in nonprofit integrity databases; and not associated with controversy or lawsuits). Organizations that pass the due diligence assessment will be scored against additional inclusion criteria (Textbox 1).

###### Veterans, Their Families, and Caregivers

We will use snowball sampling to identify potential veteran and family member participants. We will ask our network of veteran contacts (eg, the James A Haley Research Service VEG) to provide us with the names of other veterans and family members who may be interested in the MSP, using our inclusion criteria as a guide. We conducted outreach with potential veteran candidates through personal conversations with the veteran or family member to ensure a shared understanding of responsibilities, interests, and goals. Veterans and family members can decide on their own whether to participate in the MSP. The goal of recruiting veterans and family members is to establish collaborative relationships through the MSP, whereby veterans are viewed as equal partners to achieve shared goals [30].

###### Veteran CR Researchers

The ENCORE team will develop a list of researchers who have demonstrated publishing and research record focused on veteran CR. Specifically, we will identify researchers whose work has resulted in the development of CR models, policy, CR measurements, and innovations that align with the goals of ENCORE. CR researchers will be identified through a review of the veteran CR literature, and a database will be created based on the stated inclusion criteria and will include researchers who work in the VA and those from outside the VA.

##### Recruitment

ENCORE team members will recruit MSP members via email. Invitations will include the targeted language for each of the MSP stakeholder groups. Potential participants will receive a 2-week response deadline within which to respond. Up to 3 reminders will be sent within the 2-week response time frame, and the last reminder will include discrete response options: (1) yes and (2) no, not at this time.

### Phase 2 (Engage): Conduct Expert Interviews and Engage the MSP

#### Expert Informant Interviews

##### Sampling

Expert informant interviews will be conducted with representatives from VA program offices to learn about the VA’s efforts to address and provide services related to veteran CR. The goals of the interviews will be to learn how VA program offices define veteran CR, what direct or indirect services they provide to veterans, how they set priorities around veteran CR, how their VA program aligns with other VA program offices to address veteran CR, and what studies are needed to improve veteran CR services at the VA (Multimedia Appendix 1). The same inclusion criteria described in Textbox 1 for the MSP members will be used to determine which VA program offices should be contacted for interviews. For VA program offices that meet the inclusion criteria, participants for expert informant interviews will be identified by reviewing public-facing program office websites and VA intranet sites. This activity is focused on the breadth of services and programs used by the VA to support veteran CR and how research can support organizational and program priorities for great impact.

##### Recruitment

Potential expert informant interview participants will be emailed directly with an invitation to participate in a 30-minute virtual interview. Up to 3 recruitment emails will be sent. The email invitation will include the request for the person to designate another appropriate contact in their office or program if they are unable to participate.

##### Data Collection Procedures

All expert informant interviews will be conducted virtually using Microsoft Teams. Each participant will receive a short demographic questionnaire to complete and an audiovisual consent form, giving permission for the interview to be recorded. Interviews will be led by 2 experienced qualitative researchers, with one person conducting the interview using a semistructured interview guide (Multimedia Appendix 1) and the other person taking notes using a structured note-taking template. In addition, each expert informant interview participant will be asked for referrals to other VA program personnel who would have expertise relevant to the project.

##### Data Analysis

A rapid assessment process and qualitative matrix [64] will be used to analyze key informant interviews with VA program office staff. The rapid assessment process is an iterative team-based technique in which data management and analysis can occur concurrently [65]. Transcript notes will be created, which will represent the interview discussion. As a first level of analysis, notetakers will summarize these interview notes by question.

Next, a qualitative matrix will be developed using Microsoft Excel spreadsheet to organize and visualize the interview data. Transcript note summaries will be entered into the matrix according to case (rows) and questions and quotes (columns), so that in each cell, the source information is summarized as it relates to each case and question. Overall, 2 experienced qualitative team members will code the transcript note summaries using Microsoft Excel columns. Codes are words or short phrases that describe the essential meaning of textual data [66]. Qualitative consensus on the transcript note summaries and thematic descriptions will be achieved through several team meetings.

#### Engage the MSP

##### Meeting Facilitation

MSP meetings will be conducted using group facilitation techniques commonly used in organizational development and qualitative research to help groups make decisions or guide them to reach consensus [67,68]. Such techniques offer structure and leadership in group meetings without assuming authority over or responsibility for group outcomes [67,69]. Techniques include flip-charting [67,69,70], asking open-ended and probing questions, and checking in with the group to ensure that everyone is comfortable with group dynamics and processes [69]. ENCORE will collaborate with a facilitator from the Veterans Integrated Service Network 8 Organizational Development Team who is external to the project team. This facilitator will help the MSP to determine and enforce ground rules and achieve their overall and individual meeting goals and objectives.

Owing to the ongoing COVID-19 pandemic, meetings will be conducted virtually, and facilitation techniques will be modified for the virtual environment. Best practices for conducting virtual group facilitation will be identified by reviewing the literature on virtual qualitative data collection methods. Qualitative researchers have long described this approach, and increasingly, since the beginning of the COVID-19 pandemic, this approach has become more common [71-75].

**Figure 2:**
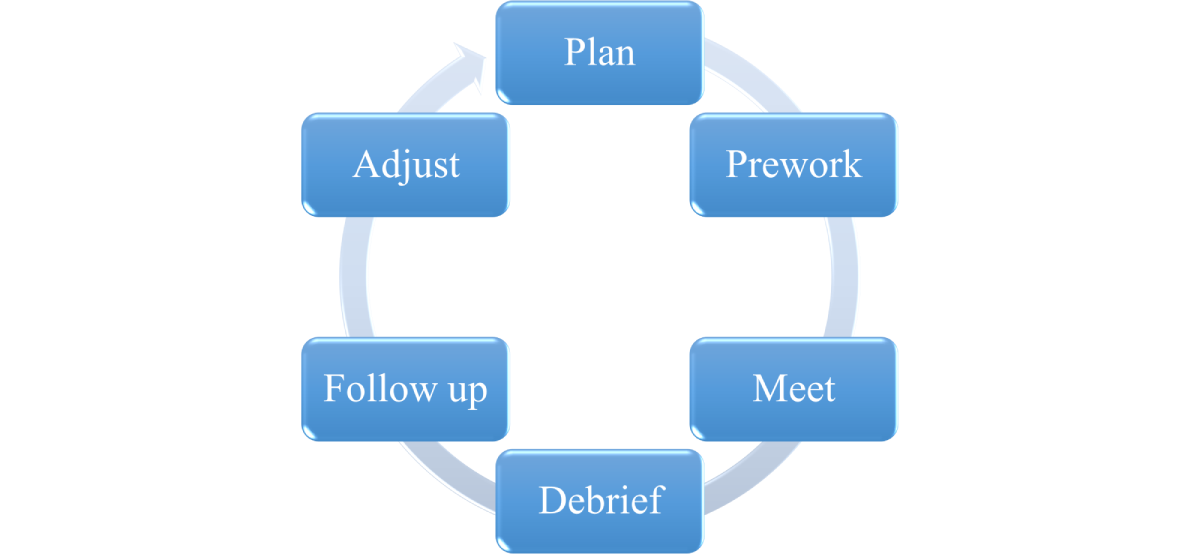
The 6-step Enhancing Veteran Community Reintegration Research Multistakeholder Partnership group facilitation process.

ENCORE will develop and use a 6-step process for conducting virtual meetings (Figure 2). This process will be developed in conjunction with a VA facilitation expert external to the team. *Step 1* is to plan. The goal of this step is to set tangible outcomes for the meeting. Here, the ENCORE team will set meeting objectives, develop an agenda, determine staff and technology needs for the meeting, identify the meeting structure (eg, Will all work be accomplished as a single large group, or will small breakout groups be required to facilitate discussion?), and gather any materials that the MSP will need to review ahead of the meeting.

*Step 2* is prework. The goal of this step will be to ensure that everyone comes to the meeting with the baseline knowledge or information that they need to accomplish the meeting goals. In this step, the ENCORE team will develop and send materials to the MSP for review; gather baseline information from the MSP by sending opinion polls and surveys to the group, if applicable; develop any presentation and breakout group materials for the meeting; and assign staff roles for the meeting. Roles will include the following: (1) facilitator, in charge of leading the meeting; (2) producer, responsible for ensuring the smooth flow of the meeting from activity to activity and troubleshooting any technology issues; (3) notetaker, in charge of observing and documenting group processes and discussions; (4) chat monitor, responsible for monitoring the chat feature of the virtual platform, responding to chats, and bringing important points to the attention of the group verbally; and (5) breakout group facilitators, in charge of facilitating small group sessions.

*Step 3* is to meet. ENCORE team members will convene half an hour before each scheduled meeting to check whether their audiovisual technology works and troubleshoot any issues. Next, a technology check will be conducted for MSP members, so that they can verify their audiovisual connections for the meeting. The group facilitator will present the goals and objectives of the meeting, review the group’s ground rules, and ensure that the group is on track to meet their goals. In some sessions, breakout groups (small groups of 4-6 MSP members and an ENCORE group facilitator and notetaker) will be needed to encourage rich discussion of a topic. After the large group reconvenes, MSP members from breakout groups will report the results of their discussion.

*Step 4* is to debrief. This critical step will directly follow the meeting during which the ENCORE team and the external facilitator will discuss what worked, what did not work, and what needs to change before the next meeting. After debriefing, *step 5* is to follow-up. During this step, the MSP will receive an *engagement and satisfaction survey* (Multimedia Appendix 2); notes from the meeting; and occasionally, a request for feedback and approval of a product developed during the meeting. This will provide the ENCORE team with an opportunity to extend engagement with the MSP. Finally, *step 6* is to adjust. The adjustment step will allow the team to synthesize information from the MSP meetings (step 3), impressions from the team debriefing (step 4), and results from the *engagement and satisfaction survey* (step 5; follow-up) to determine if and how the approach or processes need to be modified.

##### Surveys

Qualtrics [76], a web-based survey platform, will be used to survey MSP members throughout the project. Before their first meeting, MSP members will receive a *welcome survey* (Multimedia Appendix 2), which asks how they or their organization defines veteran CR, what is needed to improve veteran CR research, and demographic information. Refer to phase 5 for further information about evaluation activities.

##### Meeting Notes and Breakout Activities

A dedicated notetaker will monitor and record group processes, discussion topics, consensus, and outcomes during each meeting. Notes will be reviewed by the ENCORE team members directly following each meeting and amended, if necessary. Chat box conversations will also be included in these notes and contextualized to document every form of participation that occurs. Notes will be shared with the MSP and analyzed using rapid content analysis [77-79] to refine products, determine areas for improvement in meeting processes, and earmark items for follow-up.

### Phase 3 (Mobilize): Gap Analysis and Technical Assistance for Researchers

Gap analysis is a tool or process used to identify and define current issues within a department or field of study. Gap analysis of the relevant literature and funded veteran CR–related studies that have been published over the past 10 years will be conducted to gain a more comprehensive understanding of the state of veteran CR research. Furthermore, the areas of research on veteran CR that have received the most and least attention will be identified to highlight areas of focus that may require additional research in the future.

**Figure 3:**
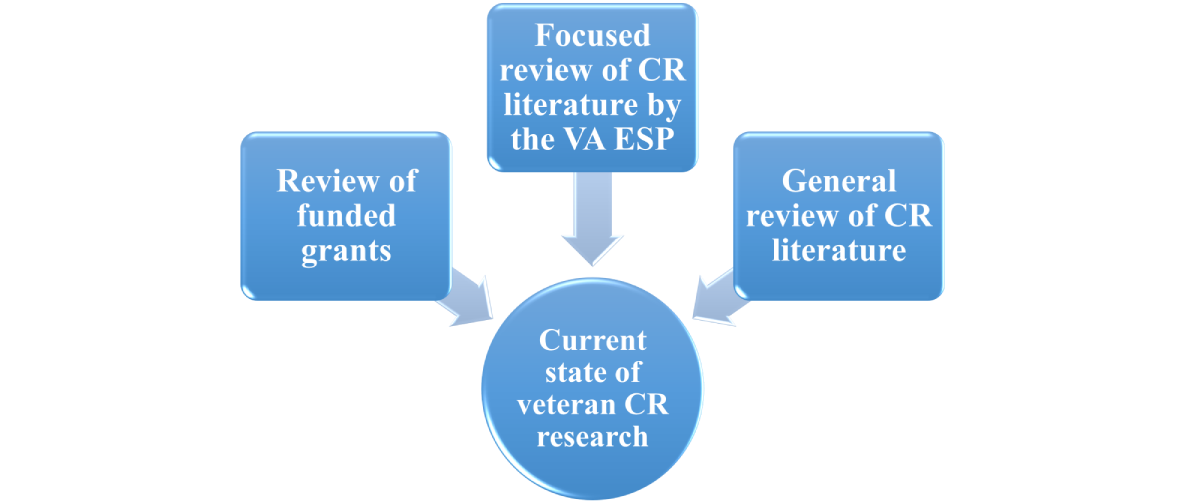
Sources contributing to the Enhancing Veteran Community Reintegration Research gap analysis. CR: community reintegration; ESP: Evidence Synthesis Program; VA: Department of Veterans Affairs.

Gap analysis of the state of veteran CR research will be framed by ENCORE’s two key veteran CR domains of focus and will use a three-step process (Figure 3): (1) review veteran CR research that was funded by VA and non-VA grants in the past 10 years; (2) collaborate with the VA ESP to compile an evidence compendium of veteran CR research focusing on interventions that promoted veteran CR among veterans with PTSD, TBI, SCI, or polytrauma; and finally, (3) review the veteran CR literature not included in the other 2 searches. Findings from research portfolio reviews, ESP requests, and literature reviews will be aligned with the 2010 RR&D SOTA goals to evaluate progress and critical gaps. This activity will form the evidence base for the research agenda–setting activities.

In years 4 and 5, ENCORE will also offer technical assistance services to researchers, VA program offices, and community organizations seeking to conduct research that is related to veteran CR. Activities and services will focus on (1) consultations that provide insight and assistance on leveraging the elements of the ENCORE veteran CR research agenda, (2) providing feedback about grant solicitations and grant proposals through the MSP meetings, and (3) connecting with experts in the field for additional support.

### Phase 4 (Promote): Communication Plan

In the *promote* phase, the ENCORE team will connect with the VA Health Services Research and Development Center for Information Dissemination and Education and consult with experts in dissemination and implementation (D&I) and KT. The primary target audiences for D&I and KT activities will be veteran CR researchers both within and outside VHA. The goal of this phase will be to communicate veteran CR research needs for great impact of research on policies and services. Through continued MSP engagement in the format of regular meetings and email communications (as described previously), a stakeholder-driven and prioritized D&I plan will be developed for ENCORE project deliverables and ENCORE-informed research, policies, and practices. Secondary audiences (eg, policy makers, service providers, and VA patients) will be targeted based on stakeholder input. In partnership with MSP stakeholders, public-facing dissemination products and resources will be developed and refined with the help of an ENCORE VEG to ensure that the products are meaningful to and tailored to meet the needs of multiple audiences. In addition, a public-facing website that contains links to selected products will be created and hosted through the Center for Information Dissemination and Education. Dissemination activities beginning in this phase, including the development of publications, are estimated to continue for at least 1 year after completing the project.

### Phase 5 (Evaluate): MSP Surveys

Following each MSP meeting, participants will receive a *meeting satisfaction and feedback survey* (Multimedia Appendix 2), asking for comments on their experiences and seeking feedback about ways to improve. Occasionally, surveys will be sent to MSP members, asking them to finalize products, make decisions, and prioritize the next steps for upcoming meetings and products. Close-ended survey items will be analyzed using descriptive statistics. Open-ended survey responses will be analyzed using a matrix analysis process [64,80]. Given the iterative nature of the project, ENCORE will review the evaluation needs regularly.

### Ethical Considerations

The local VA Research and Development Committee at the James A Haley Veterans’ Hospital has determined this evaluation to be a quality improvement project with nonresearch status. Therefore, it is not subject to human participants research ethical reviews, and written informed consent will not be required before data collection [81]. However, owing to local facility policies, written consent for audio-recording the interviews will be required for data collection.

### Privacy and Security

Interviews will be recorded using Microsoft Teams, and audio files and notes will be saved in a secured network folder. The secured folder is accessible to the members of the evaluation team. When reporting the results of the interviews, all efforts to preserve confidentiality will be made, and data will be reported in the aggregate when appropriate.

### Ensuring Inclusion and Accessibility

All recruitment and data collection materials will be drafted between eighth and tenth grade level using *plain language* principles for clear communication [82]. Technology checks and one-on-one technical assistance will be offered to ensure that all participants can fully participate in virtual meetings regardless of visual, hearing, or cognitive impairments.

## Results

As of December 2022, data collection for ENCORE is ongoing. So far, 20 representatives from 16 VA program offices providing veteran CR–related services participated in a 30-minute telephone interview. Notably, through these interviews, veteran CR was understood to occur at any time after separation, not just immediately after military separation or retirement. Furthermore, veteran CR is cyclical and extends across the veteran’s lifetime as opposed to a single point in time. ENCORE developed and assembled the MSP, reviewed the VA funding portfolio and veteran CR research literature, and conducted a scientific gap analysis. The MSP comprises VA program directors (10/25, 40%), veterans and caregivers (5/25, 20%), established veteran CR researchers (5/25, 20%), and representatives of community-based VSOs (5/25, 20%). The MSP developed a veteran CR research agenda that included 3 research priorities [22]. In addition, the MSP is currently working with the ENCORE team to prepare materials for dissemination. The ENCORE project is funded till June 2024.

## Discussion

### Significance

ENCORE’s 5-year road map contains 5 phases of activities that are essential to achieve the overall impact goal of ENCORE, which is to improve VA policies, programs, and services related to veteran CR. The ENCORE protocol engages the MSP as full partners throughout the process, including in the formation of research questions and in shaping the design, funding, conduct, and dissemination of studies [29]. Therefore, the ENCORE protocol is uniquely situated to not only identify gaps in the veteran CR research literature but also to determine a ranking of the priority of those gaps. An engaged stakeholder-driven process to generate and prioritize a research agenda is known to increase the relevance and utility of sustainable interventions and services [29,34]. Owing to the stakeholder engagement activities that ground this project and the overlapping timelines for all phases, detailed planning of each activity is critical to avoid delays and ensure that all stakeholder activities run smoothly.

Engagement is becoming understood to be central to the development, implementation, and dissemination of research activities [83]. The use of an MSP for engaging stakeholders to identify research priorities and develop a research agenda is a novel approach for mobilizing veteran CR research [84]. It differs from traditional methods for reaching consensus across sectors as a means of engagement. For example, the more traditionally used Delphi method aims to reach consensus through indirect methods such as questionnaires and typically relies on experts instead of those directly affected [85,86]. In contrast, ENCORE uses an MSP to engage stakeholders, allowing for their direct input. The MSP’s feedback will help to ensure that veteran CR research will have a lasting impact by addressing research questions identified as relevant by the veteran community [83].

### Strengths and Limitations

Strengths of this project include stakeholder engagement activities that were deliberately and thoughtfully planned to achieve project goals. A rigorous process is being used to select candidates for the MSP, and additional efforts are being made, such as using an external facilitator for well-planned and executed meetings. Given the diversity of perspectives, MSP products are more likely to be relevant and responsive. Limitations include the resources (eg, staff and time) available, which affected the project’s scope and narrowed the focus to veterans (not active duty service members); VA programs; and other governmental and nongovernmental organizations discussed by VA stakeholders, which did not include the Department of Defense. Similarly, limited resources also affected the team’s ability to plan and conduct projects that prioritize stakeholder engagement. The veteran and family member group did not constitute a representative sample of veteran viewpoints but rather a collaborative partnership where their shared voices and experiences were valued in the process of shared decision-making. In addition, owing to COVID-19, some MSP members had additional responsibilities that precluded them from attending all meetings.

### Conclusions and Implications

The goal of ENCORE is to improve the impact of veteran CR research on policies and programs. This protocol describes methods that can be used by researchers and other professionals for engaging multiple stakeholders to prioritize research, program, and policy needs of specific populations. By grounding activities in authentic stakeholder engagement to accomplish project objectives, ENCORE can work toward achieving its goal. Using a stakeholder-engaged process is more likely to result in a relevant and responsive veteran CR research program.

## Appendix

Multimedia Appendix 1 Enhancing Veteran Community Reintegration Research expert informant interview guide.

Multimedia Appendix 2 Multistakeholder Partnership questionnaires—welcome survey and engagement and satisfaction survey.

## Abbreviations

CR: community reintegration
CRIS: Community Reintegration of Injured Service Members
CRIS-CAT: Community Reintegration of Injured Service Members Computer Adaptive Test
D&I: dissemination and implementation
ENCORE: Enhancing Veteran Community Reintegration Research
ESP: Evidence Synthesis Program
KT: knowledge translation
M2CQ: Military to Civilian Questionnaire
MSP: Multistakeholder Partnership
PTSD: posttraumatic stress disorder
RR&D: Rehabilitation Research and Development
SCI: spinal cord injury
SOTA: state-of-the-art
TBI: traumatic brain injury
VA: Department of Veterans Affairs
VEG: veteran engagement group
VHA: Veterans Health Administration
VSO: veteran service organization

## Data Availability

The data sets generated and analyzed during this study are not publicly available owing to the Department of Veterans Affairs record and access policies but are available from the corresponding author upon reasonable request.
