# Supplementary material for: Enhancing Veteran Community Reintegration Research (ENCORE): Protocol for a Mixed Methods and Stakeholder Engagement Project
Source: JMIR Res Protoc. 2023 Mar 14;12:e42029. doi: 10.2196/42029 (PMC10131720; doi:10.2196/42029)
Supplement: Multimedia Appendix 1 [file resprot_v12i1e42029_app1.docx]

**Appendix 1: Enhancing Veteran Community Reintegration Research expert informant interview guide.**

| **Interview with VA Program Officers to Inform Veteran/Service Member Community Reintegration Research Agenda.** | |
| --- | --- |
| ***Our research team is funded by HSR&D (*IVI 19-487*) to help the VA improve its policies, programs and services related to Veteran community reintegration (CR) through relevant and innovative research.***  ***My*** ***goals today are to 1) understand your CR priorities from an operational standpoint and 2) understand what research is needed to help inform the [VA program office] goals relating to CR.*** | |
| Q1 | How does [VA program office] define Veteran/Service Member Community Reintegration? |
| Q2 | How does [VA program office] support Veteran/Service Member Community Reintegration? |
| Q2a/PROBE: | Which groups do these CR activities target? (any underserved veteran groups, caregivers, etc.?) |
| Q3 | Who does [VA program office] collaborate with to support Veteran/Service Member CR? |
| Q4 | What data is [VA program office] collecting about your CR services and programs? |
| Q4a/PROBE: | How will you use the data to design, improve, or implement CR services and programs? |
| Q5 | What research is needed to improve the CR services at the VA? How would you rank these needs (highest to lowest)? |
| Q6 | *Thank the participant and ask if they or a representative from their office would be interested in participating in the Multi Stakeholder Panel.* |
