# Supplementary material for: Enhancing Veteran Community Reintegration Research (ENCORE): Protocol for a Mixed Methods and Stakeholder Engagement Project
Source: JMIR Res Protoc. 2023 Mar 14;12:e42029. doi: 10.2196/42029 (PMC10131720; doi:10.2196/42029)
Supplement: Multimedia Appendix 2 [file resprot_v12i1e42029_app2.docx]

**Appendix 2: Multi-Stakeholder Partnership questionnaires – welcome survey and engagement and satisfaction survey.**

| **Multi Stakeholder Partnership (MSP) Welcome Survey** | |
| --- | --- |
| Q1  DESCRIPTIVE TEXT | ***Thank you for participating in our Multi Stakeholder Partnership (MSP). This is part of a project called Enhancing Veteran Community Reintegration Research (ENCORE). You will work with other stakeholders to set a VA research agenda about Veteran community reintegration. Prior to meeting, we want to get some baseline information from you to inform the goals and activities of the MSP.***  ***Our research team is funded by HSR&D (IVI 19-487) to help the VA improve its policies, programs and services related to Veteran community reintegration (CR) through relevant and innovative research.*** |
| Q2  TEXT | Please write your first and last name. |
| Q3  SELECT MULTIPLE | Please select all of the statements that describe you.  1, I am a Veteran.  2, I am the family member or caregiver of a Veteran. |
| Q4  TEXT | How do you define Veteran community reintegration? |
| Q5  SELECT ONE | Are you participating in the MSP as a representative from an organization that provides Veteran community reintegration services?  0, No  1, Yes – BRANCH to Q5a |
| Q5a  TEXT | How does your organization define Veteran CR?  TEXT |
| Q6  TEXT | What is needed to improve Veteran community reintegration? This might include anything: policies, programs, research, services, social supports, etc. |

| **Multi Stakeholder Partnership (MSP) Engagement and Satisfaction Survey** | |
| --- | --- |
| Q1  DESCRIPTIVE TEXT | ***You’ve just participated in the Multi Stakeholder Partnership (MSP) meeting! Now is your chance to tell us about your experience and provide feedback on what we can improve.*** |
| Q2  TEXT | Please write your first and last name. |
| Q3  SELECT ONE | The Multi-Stakeholder Partnership is a good investment of my time.  1, Agree  0, Disagree |
| Q4  SELECT ONE | We usually stayed on track during the meeting.  1, Agree  0, Disagree |
| Q5  SELECT ONE | I was given the opportunity to contribute to the discussion during the meeting.  1, Agree  0, Disagree |
| Q6  SELECT ONE | The meeting was well facilitated.  1, Agree  0, Disagree |
| Q7  SELECT ONE | During the meeting, time was taken to evaluate meeting processes.  1, Agree  0, Disagree |
| Q8  SELECT ONE | The meeting began and ended on time.  1, Agree  0, Disagree |
| Q9  SELECT ONE | Members shared responsibility to make sure the meeting was effective.  1, Agree  0, Disagree |
| Q10  SELECT ONE | We accomplished meeting objectives.  1, Agree  0, Disagree |
| Q11  SELECT ONE | The meeting was not interrupted (e.g., phones, hot mic’s, people coming and going, etc.  1, Agree  0, Disagree |
| Q12  SELECT ONE | At the end of the meeting, I was pleased by our accomplishments and ready to follow-up on action items.  1, Agree  0, Disagree |
| Q13  SELECT ONE | Overall, how would you rate this meeting?  1, Excellent,  2, Good  3, Needs improvement |
| Q14  TEXT | Which elements of the meeting worked for you? |
| Q15  TEXT | Which elements of the meeting would you change? |
| Q16  SELECT ONE | How would you rate your breakout group session?  1, Excellent  2, Good  3, Needs improvement |
| Q16a  TEXT | How could we improve the breakout group experience in the future? |
